# Effect of hyperinsulinemia during hemodialysis on the insulin-like growth factor system and inflammatory biomarkers: a randomized open-label crossover study

**DOI:** 10.1186/1471-2369-14-80

**Published:** 2013-04-04

**Authors:** Mark Reinhard, Jan Frystyk, Bente Jespersen, Mette Bjerre, Jens S Christiansen, Allan Flyvbjerg, Per Ivarsen

**Affiliations:** 1 Department of Renal Medicine, Aarhus University Hospital and Department of Clinical Medicine, Faculty of Health, Aarhus University, Aarhus, Denmark; 2 The Medical Research Laboratory, Department of Clinical Medicine, Faculty of Health, Aarhus University, Aarhus, Denmark; 3 Department of Endocrinology and Internal Medicine, Aarhus University Hospital, Aarhus, Denmark

**Keywords:** Bioactive IGF-I, Hemodialysis, IGFBP-1, Inflammation, Insulin, Nutrition

## Abstract

**Background:**

A marked reduction in serum levels of bioactive insulin-like growth factor-I (IGF-I) has been observed in fasting hemodialysis (HD) patients during a 4-h HD session. The aim of the present study was to investigate the beneficial effect of hyperinsulinemia during HD on bioactive IGF-I and inflammatory biomarkers.

**Methods:**

In a randomized cross-over study, 11 non-diabetic HD patients received a standardised HD session with either: 1) no treatment, 2) glucose infusion (10% glucose, 2.5 mL/kg/h), or 3) glucose-insulin infusion (10% glucose added 30 IU NovoRapid® per litre, 2.5 mL/kg/h). Each experiment consisted of three periods: pre-HD (−120 to 0 min), HD (0 to 240 min), and post-HD (240 to 360 min). A meal was served at baseline (−120 min); infusions were administered from baseline to 240 min. The primary outcome was change in bioactive IGF-I during the experiment. Secondary outcomes were changes in high-sensitivity C-reactive protein, interleukin-1β, interleukin-6, and tumor necrosis factor α. Comparisons were performed using mixed-model analysis of variance for repeated measures.

**Results:**

From baseline to the end of study, no significant differences were observed in the changes in either serum bioactive IGF-I or total IGF-I between study days. Overall, serum bioactive IGF-I levels rose above baseline at 120 to 300 min with a maximum increase of 20% at 120 min (95% confidence interval (CI), 9 to 31%; p < 0.001), whereas total IGF-I levels rose above baseline at 180 to 300 min with a maximum increase of 5% at 240 min (95% CI, 2 to 9%; p = 0.004). A significant difference was observed in the changes in serum IGF-binding protein-1 (IGFBP-1) between study days (p = 0.008), but differences were only significant in the post-HD period. From baseline to the end of HD, no significant difference was observed in the changes in serum IGFBP-1 levels between study days, and in this time period overall serum IGFBP-1 levels were below baseline at all time points with a maximum decrease of 51% at 180 min (95% CI, 45 to 57%; p < 0.001). None of the investigated inflammatory biomarkers showed any differences in the changes over time between study days.

**Conclusions:**

Postprandial insulin secretion stimulated the IGF-system during HD with no further effect of adding glucose or glucose-insulin infusion. Hyperinsulinemia during HD had no effect on biomarkers of inflammation.

**Trial registration:**

ClinicalTrials.gov registry: NCT01209403

## Background

Protein-energy wasting (PEW) is frequent in maintenance hemodialysis (HD) patients and an independent predictor of morbidity and mortality [1]. Causes of PEW in maintenance HD patients include reduced intake of energy and protein, resistance to the actions of anabolic hormones such as insulin, growth hormone (GH), and insulin-like growth factor-I (IGF-I) [2-5], and non-specific inflammatory processes [6-9]. Moreover, HD *per se* stimulates muscle and whole body protein loss which further increases the risk of PEW [10-12]. Amino acid kinetic studies have demonstrated that HD in the postabsorptive state induces muscle protein loss by increased muscle protein breakdown [13]. Muscle protein breakdown is stimulated by the release of proinflammatory cytokines induced by contact of blood cells with the dialyser membrane and bacterial-derived DNA fragments in the dialysis fluid [6,14,15]. Of note, there is a positive correlation between interleukin-6 (IL-6) levels and muscle protein breakdown, and as IL-6 levels peak after completion of the HD procedure a considerable protein catabolic “carry-over effect” may extend to the post-HD period [16].

IGF-I is an important regulator of muscle metabolism and stimulates muscle protein anabolism in both animals [17,18] and humans [19,20]. More than 99% of the circulating IGF-I pool is complexed to specific high-affinity IGF-binding proteins (IGFBPs) that regulate the bioavailability of IGF-I to its tissue receptors [21]. Hence, changes in the binding capacity of the IGFBPs may result in marked changes in serum free and thereby bioactive IGF-I without causing any change in serum total IGF-I [22]. Supportive of this notion, we recently reported that HD performed in overnight fasting patients resulted in a marked up-regulation of serum IGFBP-1, the only short-term regulated IGFBP, and a corresponding down-regulation of free and bioactive IGF-I without any concomitant change in total IGF-I [23]. These results may reflect the protein catabolic nature of HD in the fasting state and, moreover, they may explain why previous amino acid kinetic studies in HD patients failed to show any association between changes in muscle protein metabolism and serum total IGF-I [10,24].

The IGF-system interacts with inflammatory proteins. Experimental animal studies have demonstrated that infusion of tumor necrosis factor α (TNF-α) in rats increases the hepatic mRNA expression and protein secretion of IGFBP-1, whereas those of IGF-I are decreased [25]. In addition, in uremic rodents chronic or sepsis-induced inflammation reduces serum IGF-I levels, IGF-I gene expression, and muscle net protein synthesis; changes that can be reversed or ameliorated by pharmacological blockage of the proinflammatory cytokines IL-1, IL-6, and TNF-α [26-28].

Insulin inhibits the hepatic IGFBP-1 production and may therefore indirectly stimulate the actions of IGF-I *in vivo*[29,30]. Furthermore, insulin appears to exert anti-inflammatory effects [31,32]. Therefore, we hypothesized that maintenance of hyperinsulinemia during HD by either glucose or glucose-insulin infusion would suppress the HD-induced decrease in serum bioactive IGF-I as well as the increase in plasma IL-6.

## Methods

### Study participants

Twelve non-diabetic patients were enrolled in this study which was conducted between November 2010 and July 2011 at Aarhus University Hospital. One patient withdrew before completing the study and was omitted from data analysis. Patients included were older than 18 years, on stable maintenance HD for at least 3 months, and had well-functioning arteriovenous fistulas with recirculation less than 5%. Exclusion criteria were diabetes mellitus, body mass index (BMI) below 18.5 or above 30.0 kg/m^2^, malnutrition (subjective global assessment score C), active malignant disease, immunosuppressive treatment (including glucocorticoid treatment), evidence of an ongoing inflammatory disease, or pregnancy. Baseline characteristics are presented in Table 1. The diagnoses were chronic glomerulonephritis (n = 2), autosomal dominant polycystic kidney disease, (n = 2), medullary cystic kidney (n = 1), granulomatosis with polyangiitis (n = 1), and chronic renal failure of unknown origin (n = 5). Comorbidity was quantified according to Davies *et al.*[33]. Five patients had 0 comorbidities (low risk), 5 patients had 1–2 comorbidities (medium risk), and 1 patient had ≥3 comorbidities (high risk). Four patients smoked 10 to 20 cigarettes a day. Medication was unchanged during the study period. The study was performed in accordance with the Declaration of Helsinki and monitored by the Committee of Good Clinical Practice at Aarhus University Hospital. Protocol and consent forms were approved by the local research Ethics Committee (Central Denmark Region), and all participating patients gave written informed consent. This trial is registered with the ClinicalTrials.gov registry: NCT01209403.

**Table 1:** Patient characteristics at baseline

**Variable**	**n = 11**
Age (years)	58 (33–79)
Sex (males/females)	8/3
Body mass index (kg/m^2^)	24.9 ± 3.6
Subjective global assessment score (A/B)	5/6
Hemodialysis vintage (months)	35 (11–232)
Dialysis days per week (2/3/4 days)	1/8/2
Urine production ≥ 200 mL/day (yes/no)	4/7
Kt/V	1.58 ± 0.38
Normalized nitrogen protein appearance rate (nPNA) (g/kg/day)	0.91 ± 0.16
Plasma albumin (g/L)	38.8 ± 2.1
pH	7.43 ± 0.03
S-HCO3^-^ (mmol/L)	25.5 ± 2.0
Blood glucose (mmol/L)	5.2 ± 0.4
Serum insulin (pmol/L)	33.9 ± 19.1
Serum bioactive IGF-I (μg/L)	0.83 ± 0.27
Serum total IGF-I (μg/L)	124 ± 43
Serum IGFBP-1 (μg/L)	267 ± 147
Serum IGFBP-2 (μg/L)	1949 ± 1112

Data are mean ± SD, median (range), or number.

At baseline no differences were observed in biomarker concentrations between study days and overall mean concentrations are shown.

### Study design

In this randomized, controlled cross-over study, patients received a standardised HD session with either 1) no treatment (NT), 2) continuous intravenous infusion of glucose 10% at a rate of 2.5 mL/kg/h (G), or 3) continuous intravenous infusion of glucose 10% added 30 IU of insulin aspart (NovoRapid®, Novo Nordisk A/S, Bagsværd, Denmark) per litre at a rate of 2.5 mL/kg/h (GI). The three experiments were separated by two weeks wash-out period. In two cases the experiments were postponed for 5 days because the patients were unable to participate the scheduled day, and in one case the investigation was postponed for three weeks due to an acute upper gastrointestinal bleeding.

### Experimental study day

**Figure 1:**
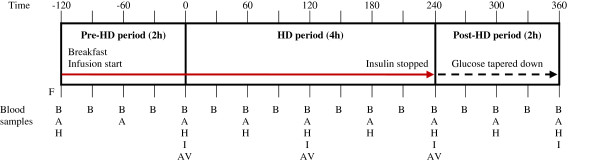
**Experimental design.** Abbreviations: F, fasting samples; B, blood glucose; A, ABL gas analyzer; H, hormones; I, inflammatory biomarkers; AV, both arterial and venous sites for insulin/insulin aspart samples.

The experimental design is depicted in Figure 1. After an overnight fast, patients met between 7:30 and 9:00 AM at the Dialysis Department at Aarhus University Hospital. Participants were instructed to meet at the same time on each of the three study days. The experiments lasted eight hours, and each experiment was divided into a pre-HD period (2 hrs), a HD period (4 hrs), and a post-HD period (2 hrs). In the pre-HD period, dialysis needles were inserted in the arteriovenous shunt and fasting blood samples collected before a standardised breakfast meal was served. The breakfast meal consisted of bread, butter, ham, cheese, jam, and honey and was served with either coffee or tea. The meal had to be ingested within the first hour of the pre-HD period and the type and amount of food ingested was registered in order to calculate intake of energy (NT: 9.2 ± 2.9; G: 8.1 ± 2.8; GI: 9.8 ± 2.9 kcal/kg) and protein (NT: 0.28 ± 0.07; G: 0.26 ± 0.06; GI: 0.31 ± 0.07 g/kg). Unintentionally, the mean energy intake was significantly higher on GI than on G study days (p = 0.007). Otherwise no significant differences in energy or protein intake were observed between study days. After the meal only sips of water and ice cubes were permitted until the end of the experiment. On the treatment days, infusions of either G or GI were commenced immediately after collection of fasting blood samples, using a second catheter inserted in the contra-lateral arm. G and GI were infused during the pre-HD and HD period and blood glucose levels (BG) were maintained at 8.0-10.0 mmol/L by additional glucose infusion. In the post-HD period, insulin infusion was stopped and glucose infusion was tapered off according to BG levels targeted at 3.0-5.0 mmol/L to avoid severe hypoglycaemia.

### Hemodialysis

HD was delivered as a standardised 4-h lasting HD session using a blood flow of 300 mL/min, a dialysate flow of 500 mL/min, and a high-flux polysulphone membrane (FX80, Fresenius Medical Care, Bad Homburg, Germany). Ultrafiltration was scheduled to be linear throughout the HD session and to compensate for the extra volume load given due to G and GI infusion to obtain previously established “estimated dry weight”. The same dose of low-molecular-weight heparin was used in all three experiments. A standardised dialysate containing glucose 5.5 mmol/L, sodium 138 mmol/L, potassium 2.0 mmol/L, calcium 1.25 mmol/L, and bicarbonate 36 mmol/L was used.

### Assays

Samples for Radiometer ABL 800 Flex blood gas analyzer (Radiometer Medical, Brønshøj, Denmark) were analyzed every hour throughout the experiment to monitor electrolytes (especially potassium), BG levels, and hematocrit. In addition, BG levels were measured with glucometer (HemoCue Glucose 201 DM, HemoCue AB. Ängelholm, Sweden) to obtain BG measurements every 30 min. In the post-HD period, BG levels were measured every 10–30 min to avoid hypoglycaemia.

Plasma concentrations of albumin, fibrinogen, and 25-hydroxy vitamin D were determined with routine automated methods at the Department of Clinical Biochemistry, Aarhus University Hospital. For measurements of hormones and inflammatory biomarkers serum and plasma were separated by centrifugation at 3500 *g* for 10 min at 4°C and aliquots were stored within an hour at −80°C until further processing.

Serum levels of immunoreactive (total) IGF-I (final dilution 1:1000) were determined in acid ethanol serum extracts using a validated, in-house time-resolved immunofluorometric assay (TR-IFMA) as previously described [34] with slight modifications: first, the IGF-I assay had been calibrated against the international IGF-I reference preparation WHO 02/254. Second, the detection antibody now consists of a biotinylated goat polyclonal antibody (Sigma-Aldrich, catalogue No. I-8773; Brøndby, Denmark). The biotinylated antibody was incubated with streptavidin europium obtained from Perkin Elmer Life Sciences (Turku, Finland).

Bioactive IGF-I was analysed by a cell-based kinase-receptor activation (KIRA) assay based on human embryonic cells transfected with cDNA of the human IGF-I receptor (IGF-IR) gene, as previously detailed [35], with slight modifications. The assay measures the ability of serum IGF to phosphorylate (i.e. activate) the IGF-IR *in vitro*, and takes into account the presence of the IGFBPs and their ability to modify IGF-IR activation by IGF-I or IGF-II [36]. In brief, transfected cells were stimulated with serum diluted 1:10 in Krebs-Ringer buffer for 15 min at 37°C, after which cells were aspirated and the cells lysed. The crude cell lysates were then transferred to a TR-IFMA assay specific for phosphorylated (i.e. ligand-activated) IGF-IRs, based on a monoclonal capture antibody against the extracellular IGF-IR domain and an europium-labelled monoclonal anti-phosphotyrosine antibody as tracer. Since the original description [35], the KIRA assay has been slightly modified. The assay has now been calibrated against the international IGF-I reference preparation WHO 02/254. Furthermore, the antibodies of the phosphorylated IGF-IR TR-IFMA have been changed. Coating is now performed using the monoclonal antibody (part 841431, 4 mg/l) contained in the human phospho-IGF-IR kit from R&D Systems (catalogue no DYC 1770E). For detection we use the biotinylated monoclonal phosphotyrosine antibody BAM 1676 (2 mg/l; R&D Systems). The latter is co-incubated with streptavidin europium.

In the KIRA assay, the ability of serum to phosphorylate the IGF-IR *in vitro* is compared of a serial dilution of rh-IGF-I (WHO 02/254), and accordingly, it is possible to express the serum signal in IGF-I concentrations. The KIRA also detects IGF-II and pro-IGF-II activation of the IGF-IR with a cross-reactivity of 12% and 2%, respectively, of that of IGF-I, whereas proinsulin, insulin and insulin analogues have a cross reactivity <1%. The KIRA has a detection limit <0.08 μg/l, and intra- and inter-assay CVs averaging <7% and <15%, respectively.

IGFBP-1 was assayed by an in-house TR-IFMA, which represents a modification of our previously described RIA [37]. In brief, microtiter plates (Nunc, Roskilde, Denmark) were coated with 100 μL per well of MAB 6303 (Medix Biochemica, Kauniainen, Finland, 1 mg/L dissolved in phosphate buffer, 40 mM, pH 8.0) and incubated overnight at 5°C. MAB 6303 recognizes all phosphorylated isoforms of human IGFBP-1 [38]. Next day, all wells were washed once (PBS, pH 7.4 added 0.5% (vol/vol) Tween 20 and 0.05% (wt/vol) NaN3) before they were blocked (40 mM phosphate buffer, pH 8.0, added 5% (vol/vol) Tween 20 and 25 μM EDTA). After 2 hrs of blocking at room temperature, the wells were washed once and added 100 μL antigen. Purified human amniotic IGFBP-1 (catalogue no 8IGB1, HyTest, Turku, Finland) served as standard (range: 1.56-50 μg/L). Serum was diluted 1:10. All antigens were dissolved in assay buffer (40 mm phosphate, pH 8.0, added 25 μM EDTA, 0.1% (wt/vol) bovine serum albumin, 0.05% (wt/vol) NaN_3_, 0.9% (wt/vol) NaCl and 1% (vol/vol) Tween 20)) and assayed in duplicates, whereas non-specific binding (NSB) was assayed in quadruplicate. After an overnight incubation at 5°C, the wells were washed and added 100 μL (400 μg/L) of polyclonal rabbit antibody directed against human IGFBP-1 (catalogue no sc-13097, Santa Cruz Biotechnology, Heidelberg, Germany) dissolved in assay buffer. After two hours of incubation at room temperature all wells were washed and added 100 μL biotinylated goat anti-rabbit IgG (catalogue no. BAF008, R&D Systems) together with Europium-labeled streptavidin added in a final dilution of 1:1000. After two hours incubation at room temperature all wells were washed 6 times, added enhancement solution (200 μL/well, Perkin Elmer Life Sciences, Turku, Finland) and read using time-resolved fluorometry. The coating antibody MAB 6303 has previously been shown to neglect rhIGF-I and -II as well as rhIGFBP-2, -3, -4, and −5 in concentrations up to 10,000 μg/L [37]. The sensitivity of the assay is well below the lowest standard (the signal to NSB ratio corresponding to the lowest and highest standards averaged approximately 7 and 70, respectively). The intra-assay CV of samples assayed in duplicates averaged less than 5%, the inter-assay CV of an internal control (IGFBP-1 standard) and a control serum sample averaged 8.1 and 7.0%, respectively (22 plates).

IGFBP-2 was assayed by an in-house TR-IFMA as previously detailed with an intra- and inter-assay CV of < 5% and < 12% respectively [37].

Serum insulin was measured by a commercially available TR-IFMA (AutoDELFIA insulin; Perkin Elmer Life Sciences). Insulin aspart (NovoRapid®) measurements were generously performed at the laboratories of Novo Nordisk A/S (Måløv, Denmark) using a homogenous immunoassay with analogue-specific antibody for insulin aspart determination.

Plasma cytokines (IL-1β, IL-6, and TNF-α) were measured in a magnetic Bio-Plex Pro Assay (Bio-Rad, Hercules, CA, USA), as described by the manufacturer. Detection limits were between 0.5 and 1 ng/L. A Luminex100 using the BioPlex Manager 6.0 software (BioRad) was used for analysis.

High-sensitivity CRP (hsCRP) was determined by an in-house TR-IFMA assay based on two commercial monoclonal antibodies (catalogue no. MAB17071 and BAM 17072 obtained from R&D Systems). The assay was performed essentially as described for the IGFBP-1 assay. As standard we used a preparation from NIBSC (1st international standard, NBISC code 85/506, Potters Bar Hertforshire, UK). The intra- and inter-assay CVs averaged less than 5 and 11%, respectively.

### Standardisation for comparison

Due to the extra volume infused on G and GI study days, the mean ultrafiltration varied between treatment days (NT: 2,398 ± 1,055; G: 3,039 ± 783; GI: 3,378 ± 645 mL), and was significantly higher on G (p = 0.003) and GI (p < 0.001) study days compared with NT study days, whereas there was no difference between G and GI study days. Despite the difference in mean ultrafiltration, the change in mean hematocrit during the experiments did not differ between study days (p = 0.72). Prior to the statistical analysis bioactive IGF-I, total IGF-I, IGFBP-1, IGFBP-2, insulin, insulin aspart, albumin, fibrinogen, hsCRP, IL-1β, IL-6, and TNF-α concentrations obtained from each patient were corrected for changes in hematocrit using the formula [39]:

$$\mathrm{corX}=X\times \frac{\left(1-Ht_{x}\right)}{\left(1-Ht_{\mathrm{baseline}}\right)}$$

where *corX* represents the corrected value for the tested compound *X*, the observed value for the tested compound, *Ht*_*x*_ the hematocrit value at the sampling time, and *Ht*_*baseline*_ the hematocrit value measured at baseline.

### Other measurements

Subjective global assessment (SGA) score was used to classify patients into three categories according to their nutritional status: A, good nutritional status; B, moderate malnutrition; C, severe malnutrition [40]. Kt/V and the normalized nitrogen protein appearance rate were evaluated by the urea kinetic model. The percentage removal fraction (RF) of serum insulin and insulin aspart by the dialysis membrane was calculated at the beginning (t = 0 min), midway (t = 120 min), and at the end (t = 240 min) of the HD session from the geometric mean ratio between insulin and insulin aspart concentrations at the arterial and venous sites of the dialyser.

### Statistical analysis

Data are expressed as the mean ± standard deviation (SD), unless otherwise stated. Changes in biochemical markers over time between and within the different study days (NT, G, and GI) were tested after logarithmic transformation of the original data with mixed-model analysis of variance (ANOVA) for repeated measures with Greenhouse-Geisser epsilon correction. In the ANOVA models, time was the repeated factor, individuals and their interaction with day of visit were random factors; order of study days, day of visit, study day, time, and the interaction between study day and time were fixed factors. When there was a significant interaction between study day and time, study days were compared pairwise, and finally differences between non-parallel curves were assessed at each time point. When no significant interaction was identified between study day and time, results from the three study days were pooled and main effects of time on overall concentrations of the biochemical marker evaluated. The percentage change from baseline was calculated from the geometric mean ratio between concentrations at baseline and at the specific time point obtained by taking antilog to the differences derived from the ANOVA models (in a log-scale). Normality was checked by QQ-plots of the residuals. Changes in insulin and insulin aspart concentrations between the arterial and venous sites of the dialyser were assessed by Student’s paired *t*-test. The percentage removal fraction (RF) of circulating serum insulin by the dialysis membrane was estimated from the geometric mean ratio between insulin concentrations at the arterial and venous sites obtained by taking antilog to the estimated difference between means of log-transformed insulin concentrations at the arterial and venous sites. Assumptions were assessed with QQ-plots of differences and Bland-Altman plots. Associations between variables were assessed with the random coefficient model and expressed as the slope coefficient between the variables. Residuals were assessed graphically and logarithm transformation of the exposure variable was performed if needed. P-values ≤ 0.05 were considered statistically significant. Data were analysed using Stata version 11.1 (Lakeway Drive, Texas, USA).

### Sample size calculation

In a previous study on 10 non-diabetic HD patients we found the mean concentrations ± SD of bioactive IGF-I to be 1.07 ± 0.41 μg/L. A difference of 20% (0.2 × 1.07 = 0.21 μg/L) was considered to be the relevant difference between study days. With a level of significance of 5% and a power of 80%, 10 subjects needed to be included in the study as the SD of the difference was estimated to be half the SD of the population SD. In order to randomize subjects equally between order of study days (6 possible combinations) and allow for drop-outs, 12 subjects were included in the study. Randomization was done in two blocks and each block contained the 6 different combinations of study days.

## Results

### Blood glucose, glucose infusion rates, and serum insulin

**Figure 2:**
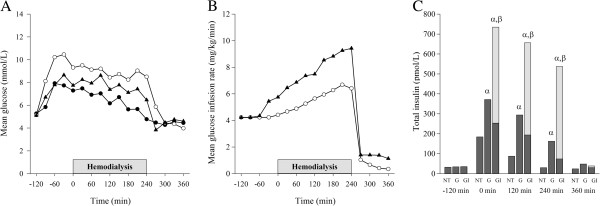
**Blood glucose and serum insulin levels.** Blood glucose levels (**A**), glucose infusion rates (**B**), and serum total insulin levels (**C**) during the experiments on no treatment (NT) (black circles), glucose infusion (G) (open circles), and glucose-insulin infusion (GI) (black triangles) study days. In plot C dark bars equal insulin and light bars equal insulin aspart levels. ^α^p < 0.001 vs NT and ^β^p < 0.001 vs G study days at same time point. Data are expressed as means.

Figure 2 depicts BG levels, glucose infusion rates, and serum total insulin (insulin + insulin aspart) concentrations on study days. Mean BG levels were comparable at baseline (NT: 5.3 ± 0.4; G: 5.1 ± 0.5; GI: 5.1 ± 0.4 mmol/L, p = 0.13), but a significant difference in the changes over time were observed between study days (p < 0.001). During the experiments, BG levels were higher on G (p < 0.001) and GI (p = 0.02) study days compared with NT study days. BG levels were lower on GI than on G study days (p = 0.01) although the total amount of infused glucose was significantly higher on GI than on G study days (2.61 ± 0.23 vs 1.94 ± 0.22 g/kg, p < 0.001). On GI study days, a pronounced decline in BG levels was observed immediately after glucose infusions were tapered off after the HD session. Hence, 30 min post-HD BG levels reached their nadir values on GI study days and were significantly lower than on G study days (3.8 ± 1.3 vs 5.7 ± 1.1 mmol/L, p < 0.001). Total insulin concentrations differed significantly between study days at 0, 120, and 240 min (p < 0.001 for each pairwise comparison), whereas no differences were observed at baseline or at 360 min.

### Serum bioactive IGF-I and total IGF-I

**Figure 3:**
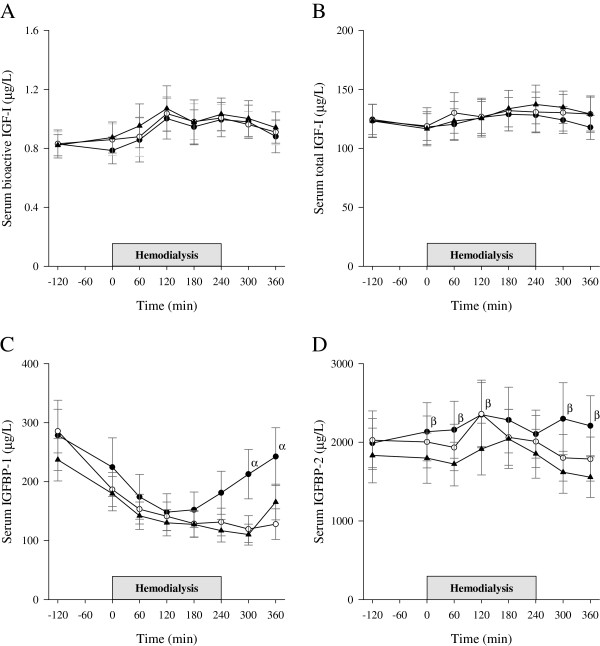
**Serum bioactive IGF-I, total IGF-I, IGFBP-1, and IGFBP-2.** Bioactive IGF-I (**A**), total IGF-I (**B**), IGFBP-1 (**C**), and IGFBP-2 (**D**) changes during the experiments on no treatment (black circles), glucose infusion (open circles), and glucose-insulin infusion (black triangles) study days. ^α^p < 0.003 for NT vs G and ^β^p < 0.02 for NT vs GI study days. Data are expressed as mean ± SEM.

No significant differences were observed in the changes over time in either serum bioactive IGF-I or total IGF-I between study days (Figure 3). Overall, postprandial serum levels of bioactive IGF-I rose above baseline at 120 to 300 min (p < 0.014 for each) with a maximum increase of 20% at 120 min (95% confidence interval (CI), 9 to 31%; p < 0.001). Overall, serum total IGF-I levels showed only modest deviations from baseline during the experiments. After a transient fall of 6% at 0 min (95% CI, 3 to 9%; p < 0.001), total IGF-I levels rose above baseline at 180 to 300 min with a maximum increase of 5% at 240 min (95% CI, 2 to 9%; p = 0.004). Bioactive IGF-I was positively related with the logarithm of total IGF-I (p < 0.001, slope coefficient = 0.59) and with the logarithm of total insulin (p = 0.03, slope coefficient = 0.02), and negatively associated with the logarithm of IGFBP-1 (p < 0.001, slope coefficient = − 0.12).

### Serum IGFBP-1 and IGFBP-2

A significant difference was observed in the changes in serum IGFBP-1 between study days (p = 0.008) (Figure 3). Differences were only found between NT and G study days and restricted to the post-HD period, where IGFBP-1 levels were higher on NT than on G study days (p < 0.003 at 300 and 360 min). From baseline to the end of HD, no significant differences were observed in the changes in serum IGFBP-1 levels between study days (p = 0.25), and in this time period overall serum IGFBP-1 levels were below baseline at all time points (p < 0.001 for each) with a maximum decrease of 51% at 180 min (95% CI, 45 to 57%). A significant difference was observed in the changes in serum IGFBP-2 between study days (p = 0.036), and further evaluation showed a difference between NT and GI study days (p = 0.013) (Figure 3). IGFBP-1 was negatively related with the logarithm of total insulin (p < 0.001, slope coefficient = − 22), whereas the same was not observed for IGFBP-2.

### Present findings compared with previous findings on fasting HD patients

**Figure 4:**
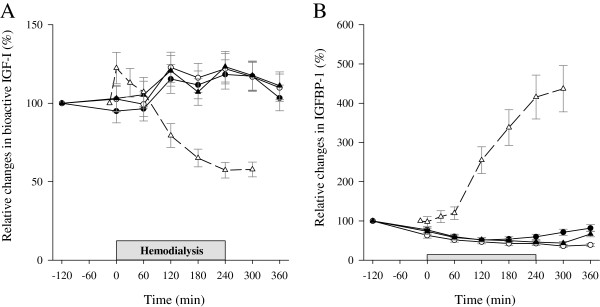
**Relative changes in serum bioactive IGF-I and IGFBP-1.** Relative changes in bioactive IGF-I (**A**) and IGFBP-1 (**B**) on no treatment (black circles), glucose infusion (open circles), and glucose-insulin infusion (black triangles) study days in the present study compared with relative changes in our previous study on fasting patients (empty triangles and dashed line). Data are expressed as mean ± SEM.

In the present study all subjects received a meal prior to HD, and they also received a glucose-containing dialysate. By contrast, in our former study we investigated ten overnight fasted non-diabetic HD patients, who remained fasting throughout the entire HD session. Furthermore, in that study we used a glucose-free dialysate, but otherwise we used a similar HD setting (4-h HD sessions using a high-flux membrane) and a similar patient group (male/female: 8/2, mean age 52.5 ± 5.7 years, BMI 26.2 ± 1.3 kg/m^2^, and no signs of inflammation or malnutrition) [23] as in the present study. Thus, by combining the two studies, we have been able to compare the impact of fasting (no meal and the use of glucose-free dialysate) with the impact of a meal and a glucose containing dialysate. The results are depicted in Figure 4. In the fasting study bioactive IGF-I was reduced by 40%, whereas IGFBP-1 increased by 5-fold. Total IGF-I levels remained stable at all time points during HD compared with baseline, but declined 11% after the HD session (from 180 ± 61 μg/L at baseline to 161 ± 63 μg/L at 300 min, p < 0.01; data not shown).

### Removal fraction of insulin and insulin aspart

As depicted in Table 2, a marked reduction in serum concentrations of both insulin and insulin aspart were observed from the arterial site to the venous site of the dialyser at the beginning (0 min), midway (120 min), and at the end (240 min) of the HD session (p < 0.001 for each comparison). The removal fraction did not differ between study days and were similar for insulin and insulin aspart. The removal fraction, however, declined from the beginning to midway of the HD session for both insulin (p < 0.001, overall) and for insulin aspart (p = 0.006), but then stayed constant until the end of the HD session.

**Table 2:** Removal fraction of insulin and insulin aspart

**Time point**	**Study day**	**A-site**	V-site*	RF**
		**(pmol/L)**	**(pmol/L)**	**(%)**
0 min	NT [insulin]	184 ± 91	57 ± 37	75 (58; 85)
	G [insulin]	371 ± 274	119 ± 100	70 (63; 76)
	GI [insulin]	253 ± 212	85 ± 91	72 (62; 79)
	GI [insulin aspart]	481 ± 227	155 ± 56	67 (56; 75)
120 min	NT [insulin]	87 ± 63	40 ± 30	55 (50; 59)
	G [insulin]	294 ± 237	146 ± 133	53 (49; 56)
	GI [insulin]	194 ± 163	94 ± 89	55 (49; 59)
	GI [insulin aspart]	462 ± 181	225 ± 75	50 (45; 56)
240 min	NT [insulin]	30 ± 20	17 ± 14	52 (32; 67)
	G [insulin]	162 ± 115	90 ± 70	47 (44; 51)
	GI [insulin]	74 ± 41	39 ± 22	48 (38; 56)
	GI [insulin aspart]	464 ± 159	250 ± 114	48 (39; 55)

Abbreviations: NT, no treatment; G, glucose infusion; GI, glucose-insulin infusion; A-site, arterial site; V-site, venous site of the dialyser; RF, removal fraction.

*P < 0.001 for comparison of insulin and insulin aspart concentrations between the A- and V-site at each time point and study day.

**The RF of insulin did not differ between study days and were similar for insulin and insulin aspart. From 0 to 120 min the RF declined for both insulin (p < 0.001, overall) and insulin aspart (p = 0.006), whereas the changes in the RF from 120 to 240 min did not reach statistical significance. Data are expressed as mean ± SD for insulin concentrations and mean (95% confidence interval) for RF.

### Inflammatory biomarkers

Changes in inflammatory biomarkers from HD start to 2 h post-HD (360 min) are depicted in Table 3. None of the investigated biomarkers showed any differences in the changes over time between study days. Compared with the start of HD there was a significant overall increase in both plasma albumin and fibrinogen concentrations at 360 min (p < 0.001 for both). From HD start to the end of the study, no changes were observed in overall concentrations of hsCRP, IL-6, or TNF-α, whereas overall concentrations of plasma IL-1β declined (p = 0.002).

**Table 3:** Changes in inflammatory biomarkers during the experiments

**Inflammatory biomarkers**	**HD start**		**End of study**		**p**^**a**^	**p**^**b**^
	**(0 min)**		**(360 min)**			
P-Albumin (g/L)	Overall:	38.8 ± 2.1	Overall:	40.4 ± 2.5	0.12	< 0.001
Mean ± SD	NT:	39.5 ± 1.4	NT:	41.3 ± 2.3		
	G:	38.1 ± 1.9	G:	40.4 ± 2.7		
	GI:	38.6 ± 2.8	GI:	39.6 ± 2.4		
P-Fibrinogen (μmol/L)	Overall:	11.7 ± 1.7	Overall:	12.8 ± 1.8	0.12	< 0.001
Mean ± SD	NT:	11.9 ± 1.9	NT:	13.0 ± 2.0		
	G:	11.6 ± 1.6	G:	12.6 ± 1.6		
	GI:	11.7 ± 1.9	GI:	12.8 ± 2.0		
S-hsCRP (mg/L)	Overall:	4.52 [0.40-10.68]	Overall:	4.67 [0.44-11.25]	0.61	0.55
Median [range]	NT:	4.75 [0.58-10.08]	NT:	6.06 [0.55-11.25]		
	G:	3.21 [0.50-8.20]	G:	3.35 [0.57-7.96]		
	GI:	6.48 [0.40-10.68]	GI:	6.40 [0.44-10.23]		
P-IL-1β (ng/L)	Overall:	0.042 [0.010-0.185]	Overall:	0.039 [0.010-0.095]	0.68	0.002
Median [range]	NT:	0.042 [0.011-0.185]	NT:	0.031 [0.011-0.049]		
	G:	0.041 [0.010-0.157]	G:	0.039 [0.010-0.095]		
	GI:	0.043 [0.010-0.166]	GI:	0.039 [0.029-0.079]		
P-IL-6 (ng/L)	Overall:	0.19 [0.04-2.33]	Overall:	0.18 [0.02-0.89]	0.96	0.25
Median [range]	NT:	0.14 [0.08-1.84]	NT:	0.19 [0.04-0.89]		
	G:	0.21 [0.04-0.97]	G:	0.17 [0.05-0.34]		
	GI:	0.20 [0.06-2.33]	GI:	0.30 [0.02-0.45]		
P-TNF-α (ng/L)	Overall:	0.42 [0.02-9.36]	Overall:	0.29 [0.02-1.82]	0.76	0.09
Median [range]	NT:	0.27 [0.10-8.53]	NT:	0.27 [0.02-1.82]		
	G:	0.51 [0.02-2.89]	G:	0.26 [0.03-1.75]		
	GI:	0.36 [0.25-9.36]	GI:	0.46 [0.25-1.33]		

Abbreviations: HD, hemodialysis; NT, no treatment; G, glucose infusion; GI, glucose-insulin infusion; hsCRP, High-sensitivity CRP; IL-1β, interleukin-1β; IL-6, interleukin-6; TNF-α, tumor necrosis factor α.

^a^p-value for comparison of differences in the change over time between no treatment (NT), glucose infusion (G), and glucose-insulin infusion (GI) study days.

^b^p-value for comparison of overall change in biomarkers from HD start to the end of the study.

## Discussion

The present study investigated the effect of maintaining hyperinsulinemia during HD by either glucose or glucose-insulin infusion on serum bioactive IGF-I and plasma IL-6. Although much higher serum insulin levels were obtained when glucose or glucose-insulin infusion was added to the meal, this had no further impact on serum levels of bioactive IGF-I or IGFBP-1 during HD than the meal alone. In contrast to the postprandial intradialytic rise in bioactive IGF-I and concomitant fall in IGFBP-1 observed in the present study, we previously reported that HD in the fasting state results in a marked reduction in bioactive IGF-I and a five-fold increase in IGFBP-1 [23]. The observations in our previous study were explained by the fasting state of the patients and the use of a glucose-free dialysate resulting in very low insulin levels (< 10 pM) during the HD sessions. Taken together, postprandial insulin secretion blocked hepatic IGFBP-1 production and stimulated serum bioactive IGF-I with no further effect of adding glucose or glucose-insulin infusion to the meal. On the other hand, although no differences in serum bioactive IGF-I were observed between study days, our two studies have demonstrated that in non-diabetic HD patients a meal can prevent the HD-associated negative changes in the IGF system observed under fasting conditions. Interestingly, protein turnover studies have almost unanimously demonstrated that intradialytic nutritional supplementation, administered either parenterally or orally, prevent the HD-associated muscle protein catabolism observed under fasting conditions and result in an anabolic muscle protein response [24,41,42].

In healthy individuals circulating IGFBP-1 levels are inversely correlated with serum insulin levels, and serum insulin levels between 65 and 172 pmol/L promptly inhibits the hepatic IGFBP-1 production [29,43]. In accordance with this, the present study demonstrated a significant negative association between serum IGFBP-1 and insulin levels. Moreover, on NT study days IGFBP-1 reached nadir values and began to rise when postprandial serum insulin levels dropped below 100 pmol/L at 120 min (~4 h after the meal). In contrast, serum IGFBP-1 concentrations were suppressed during the G and GI study days until the post-HD period when glucose infusions were tapered off, resulting in reduced insulin levels. The rise in IGFBP-1 levels 3–4 h after the meal on NT study days is similar to the postprandial response reported in healthy individuals, hereby supporting that the normal feedback of insulin on IGFBP-1 is preserved in HD patients [29,44,45]. Most likely, the steep rise in IGFBP-1 levels observed in the post-HD period on GI study days was caused by the rapid and pronounced decline in BG levels blocking the secretion of endogenous insulin and thereby stimulating IGFBP-1 secretion [46].

Insulin plays a central role in muscle metabolism both directly by inhibiting muscle proteolysis [5,20] and indirectly through the IGF-system. Insulin has a molecular weight (MW) of 5.8 kDa and usually circulates in its free form explaining that it is almost completely cleared from the blood during HD [39,47]. In contrast, more than 99% of IGF-I (MW 7.5 kDa) circulates bound to IGFBPs and therefore avoids clearance by the dialyser [23,39]. In one report, insulin was undetectable in the filtrate despite a high elimination rate and it was suggested that insulin was cleared from the blood by adsorption onto the dialyser membrane (a polysulfone membrane) [48]. In our study, we used a high-flux polysulphone membrane, which is reported to have a higher clearance rate of insulin than other membrane types [47]. During HD, the RF declined for both insulin and insulin aspart. Although speculative, this observation can be explained by saturation of the insulin binding sites on the polysulphone membrane during the HD session. Even though serum insulin levels were adequately raised by the meal, the high RF of insulin may still play a role for HD-associated muscle protein catabolism in diabetics without a residual β-cell function. In most European countries meals are routinely offered as an integrated part of the HD treatment. In contrast, some dialysis clinics in the USA have rules against meal intake during HD due to the risk of postprandial hypotension, choking/aspiration, and infection [49].

In the present study, no anti-inflammatory effect of insulin was detected as none of the investigated inflammatory markers showed any difference in the change over time between study days. In comparison, a recent cross-over study showed that compared with a 4-h HD session conducted during fasting but preceded by a breakfast meal, the addition of low-dose insulin infusion (Actrapid 2 IU/h) during HD resulted in a reduction in plasma CRP, whereas IL-6 and TNF-α levels were unaffected [32].

With respect to overall changes in acute-phase reactants, we found a highly significant increase in overall plasma albumin and fibrinogen from HD start to 2 hrs post-HD, whereas serum levels of hsCRP did not change. The rise in plasma albumin is counterintuitive since HD is an inflammatory event and albumin is considered a negative acute-phase reactant. The finding is nevertheless consistent with published literature, in which a coordinated increase in the hepatic fractional synthesis rates of albumin and fibrinogen in response to HD has been reported, and presumed to be mediated by an intradialytic rise in IL-6 and by amino acids derived from muscle catabolism [15,16,50,51].

Previous reports on changes in CRP levels during HD are inconsistent as some studies show an increase [32,51,52] and others no change [6,16,53]. The concentrations of IL-6 and TNF-α were unchanged in our study, whereas IL-1β concentrations fell. This is in contrast to previous studies that almost unanimously have reported a rise in IL-6 levels and either no change or an increase in IL-1β and TNF-α in response to HD [15,16,51,53]. The discrepancy in plasma IL-6 changes during HD may in part be explained by differences in patient characteristics as patients included in our study were well-nourished and showed no sign of infection or inflammation.

A limitation of this study is that we did not include a study day, where patients were kept fasting during the entire experiment. Therefore we compared the results of the present study with the results of a study published in 2010, where patients were in the fasting state before and during the HD session. The two studies were conducted at the same hemodialysis department and were comparable with respect to patient groups and delivered HD dose. Nevertheless, the comparisons have to be interpreted with caution. First of all, although the two patient groups were similar in the two studies the between-group variation is much greater than in a cross-over study and therefore the power to detect a difference is smaller. Secondly, although samples were analyzed at the same laboratory they were not analyzed simultaneously. Thirdly, hemodialysis procedures changed in the time period between the two studies and therefore we used different high-flux membranes (FX80, Fresenius Medical Care, Bad Homburg, Germany versus Polyflux H17, Gambro, Sweden) and dialysate compositions (glucose containing versus non-glucose containing). Despite this reservation, the differences in the changes in serum levels of bioactive IGF-I and IGFBP-1 between the two studies were of such a magnitude that we find it highly unlikely that they were caused solely by the above mentioned study differences. Finally, the patients included in the present study were non-diabetic and moderately to well nourished. It is therefore possible that results are not applicable to the general HD patient population, which also includes patients with diabetes as well as malnourishment.

## Conclusions

Adding glucose or glucose-insulin infusion to a meal had no further stimulating effects on serum IGF-I during a HD session. The study, however, demonstrated that a meal alone changed the negative response observed in the IGF-I system during HD in the fasting state to a positive response with a rise in bioactive IGF-I. These changes in bioactive IGF-I during HD support the anabolic role of intradialytic nutritional support. Hyperinsulinemia during HD had no effect on biomarkers of inflammation.

## Competing interests

The authors declare that they have no competing interests.

## Authors’ contributions

MR participated in the design of the study, carried out the experiments, performed the statistical analysis, and drafted the manuscript. JF and PI conceived of the study and its design, supervised the conduct of the experiments, helped to interpret the data, and drafted the manuscript. MB carried out the cytokine measurements, and contributed to drafting of the manuscript. BJ, JSC and AF participated in the design of the study and critically revised the manuscript. All authors read and approved the final manuscript.

## Pre-publication history

The pre-publication history for this paper can be accessed here:

http://www.biomedcentral.com/1471-2369/14/80/prepub
